# The Significance of Planned Fertility Preservation for Women With Endometrioma Before an Expected Ovarian Cystectomy

**DOI:** 10.3389/fendo.2021.794117

**Published:** 2021-12-15

**Authors:** Yeon Hee Hong, Hyun Kyoung Lee, Seul Ki Kim, Jung Ryeol Lee, Chang Suk Suh

**Affiliations:** ^1^ Department of Obstetrics and Gynecology, Seoul National University Bundang Hospital, Seongnam, South Korea; ^2^ Health Promotion Center, Seoul National University Bundang Hospital, Seongnam, South Korea; ^3^ Department of Obstetrics and Gynecology, Seoul National University College of Medicine, Seoul, South Korea; ^4^ Department of Surgical Oncology, Sheikh Khalifa Specialty Hospital, Ras Al Khaimah, United Arab Emirates

**Keywords:** cryopreservation, endometrioma, fertility preservation, ovarian cystectomy, ovarian reserve

## Abstract

Endometrioma is known to reduce the ovarian reserve and the extent of the decrease is more severe when ovarian surgery is performed. Therefore, to prevent this decline in fertility, patients with endometrioma are considered candidates for preoperative fertility preservation (FP). In this study, we evaluate the efficacy of FP in women with endometrioma before planned ovarian surgery. A total of 95 cycles in 62 patients with endometrioma, undergoing controlled ovarian stimulation (COS) for FP using a gonadotropin-releasing hormone (GnRH) antagonist protocol before an expected ovarian surgery, were enrolled retrospectively. COS outcomes were compared according to endometrioma laterality. Additionally, first COS cycle outcomes in patients with endometrioma were compared with those in infertile patients, or in patients with a benign ovarian cyst using propensity score matching. When multiple COS cycles were performed, the results of cumulative cycles were analyzed. Embryo quality was worse in the bilateral endometrioma group. Compared with the infertile patient group, the patients with endometrioma had significantly lower Anti-Müllerian Hormone (AMH) and fewer numbers of oocytes retrieved (median, 3.3 vs. 1.2, p<0.001; 7.0 vs. 4.0, p=0.009, respectively). Compared with mature oocytes in infertile patients or patients with a benign cyst, mature oocytes were fewer in patients with endometrioma, but this was not statistically significant (median, 4.0 vs. 3.0, p=0.085; 5.5 vs. 3.0, p=0.052, respectively). The median value of the cumulative number of cryopreserved oocytes or embryos was 14.5 up to the fourth cycle compared to 3 up to the first cycle, with cumulative effect. Women with endometrioma should be counseled for FP before planned ovarian cystectomy. The number of cryopreserved oocytes or embryos can be increased by repeated cycles.

## Introduction

Endometriosis is a chronic inflammatory disease in which endometrial glands are present outside the uterus, causing dysmenorrhea, pelvic pain, and dyspareunia (1). It has been reported that endometriosis affects up to 10% of women of childbearing age and up to 50% of women with infertility (2). Endometriosis also directly threatens fertility. In affected women, endometriosis can continue to aggravate symptoms and cause infertility, if left untreated. Therefore, if a patient is diagnosed with endometriosis and she intends to become pregnant or desires to become pregnant in the future, it is necessary to preserve her fertility.

Endometrioma, a type of endometriosis occurring in the ovary, decreases fertility by impaired oocyte capture due to changes in pelvic structure, impaired sperm movement, inhibited embryo development due to chronic inflammatory conditions, and negative effects on the endometrium (1, 3). In addition, endometrioma is known to reduce the primordial follicle pool by exposing healthy ovarian tissue to free radicals and decreasing the ovarian reserve by inducing mechanical stretching (4, 5). If pain is severe, the endometrioma is large, or malignancy is suspected, surgical treatment is indicated. However, ovarian surgery lowers anti-Müllerian hormone (AMH) levels (6–8) and antral follicle count (AFC) (9, 10) and the extent of the decrease is more severe when both ovaries are affected (11). Therefore, to prevent this decline in fertility, patients with endometrioma are considered candidates for preoperative fertility preservation (FP).

There is limited data on FP before ovarian surgery in patients with endometrioma (12), with few articles analyzing FP outcomes. The case report on oocyte cryopreservation in a patient with endometriosis published in 2009 is the first case of FP (13). A systematic review paper, excluding reviews and animal experiments, yielded only two case reports, one histological science study, and four retrospective cohort studies for endometriosis and FP (14). However, these studies had limitations such as being presented as a case report (13), describing a single case of tissue cryopreservation (15), involving only a few patients (16–18), involving inconsistent AMH measurement methods (19), grouping endometriosis patients as non-oncologic patients (18, 20), or involving a comparison between surgical and nonsurgical groups among patients with endometrioma without the inclusion of healthy controls (17). Since then, only a few articles have been published in 2020, including our previous paper (21–23). In addition, ovarian cysts other than endometriomas also require surgical treatment when they are large, symptomatic, or when pathologic confirmation is required. In this study, we aimed to expand the scope of the previous report on oocyte cryopreservation (21) by analyzing more patients with endometrioma and by comparing the efficacy of oocyte or embryo cryopreservation between patients with endometrioma and patients with other ovarian cysts that require preoperative surgery or infertile patients without endometrioma.

The preservation of fertility in women with endometrioma is especially important, but reliable data is lacking. In this study, we demonstrated the effect of endometrioma on controlled ovarian stimulation (COS) outcomes and verified the efficacy of FP in women with endometrioma scheduled for ovarian cystectomy.

## Materials and Methods

### Subjects

The study populations were patients with endometrioma. The patients were enrolled retrospectively from May 2016 to March 2020 at the Seoul National University Bundang Hospital. The study protocol was approved by the Institutional Review Board of the Seoul National University Bundang Hospital (No. B-2006/616-108). Patient information was obtained from electronic medical records. The condition of the enrolled patients satisfied the following three criteria: presence of endometrioma confirmed by ultrasonography (USG) and/or magnetic resonance imaging (MRI), presence of symptoms or the need for surgery owing to an increase in cyst size, and history of cryopreservation of oocyte or embryo cryopreservation for FP before ovarian surgery. On USG, endometrioma showed typical features of ground glass echogenicity (24). MRI was performed to aid diagnosis, where the features of endometrioma were diverse. Typically, confirming the shading sign as a pathognomonic feature in T2-weighted images, high signal intensity in T1-weighted images, or the presence of thick fibrous capsules, which is the most characteristic pathologic feature, are the specific features of endometrioma on MRI (25). Elective FP was performed after counseling if the preoperative AMH (Elecsys assay, Roche Diagnostics, Switzerland) value was less than 3.0 ng/mL, recurrent endometrioma was present, bilateral endometrioma were noted, or at the patient’s request. In cases of oocyte or embryo cryopreservation, the target number of cryopreserved oocytes or embryos was ≥10. If this number was not achieved after one trial, an additional cycle was performed, with the patient’s consent. Before surgery, oocyte cryopreservation was performed for unmarried women, and either embryo or oocyte cryopreservation was performed for married women.

The control groups were either infertile patients without endometrioma, who underwent *in vitro* fertilization/intracytoplasmic sperm injection – embryo transfer (IVF/ICSI-ET) or patients with a benign cyst. Infertile patients are those who underwent IVF/ICSI-ET owing to male factor, tubal factor, or an unexplained factor. For comparison of infertile patients with patients with endometrioma, propensity scores were calculated based on age and body mass index (BMI). Infertile patients and the endometrioma patients were matched 1:1.

Other benign cysts were also diagnosed *via* USG. These included mainly mature cystic teratomas, serous cystadenomas and mucinous cystadenomas. Mature cystic teratoma presents posterior sonic shadowing, diffuse or focal hyperechogenicity, hyperechoic lines or dots, and fat-fluid levels in USG (26). Serous cystadenoma presents as complex thin-walled, uni- or multilocular cysts, with variable sizes. The cyst contents show echogenic materials, possibly with or without papillary projections. Mucinous cystadenoma reveals echogenic, thin-walled multiloculated cystic mass, with various echographic presentations (27). Patients with a benign cyst and the patient with endometrioma were matched 1:1 with propensity score matching, and further analysis with linear regression analysis due to the small number of cases.

### The Controlled Ovarian Stimulation Protocol and Cryopreservation of Oocytes and Embryos

The basic COS protocol is similar to that of a previous study (21). Briefly, gonadotropin-releasing hormone (GnRH) antagonist protocols were applied to all COS cycles in endometrioma. COS was performed using either a recombinant FSH (rFSH; Gonal-F; Merck Serono, Geneva, Switzerland), or human menopausal gonadotropin (hMG; IVF-M; LG, Korea). The starting dose of gonadotropin was selected according to the patient’s age and AMH level. When the leading follicle reached a mean diameter of 13-14 mm, GnRH antagonist (Cetrotide, 0.25 mg; Serono, Darmstadt, Germany) was added to inhibit premature LH surge. When two or more leading follicles reached a mean diameter of ≥18 mm, either 250 μg of recombinant human chorionic gonadotropin (rhCG, Ovidrel; Merck Serono, Darmstadt, Germany) or 250 μg of rhCG plus a GnRH agonist (0.2 mg of Decapeptyl; Ferring) was administered for final oocyte maturation. Oocyte pickup was performed 36 hours after triggering, under ultrasound guidance. After retrieval, the oocytes were evaluated by two expert embryologists. Only mature oocytes which had a polar body in the perivitelline space were cryopreserved through the vitrification method. The same COS protocol was applied for infertile patients and patients with other ovarian cysts. In cases of embryo cryopreservation, IVF or ICSI was performed 4 h after oocyte retrieval and then cryopreserved at either cleavage or blastocyst stage.

The basal characteristics were compared according to the total number of patients (n=62). All COS results were analyzed as total FP cycles (n=95), considering each cycle as independent of oocyte cryopreservation (n=77) and embryo cryopreservation (n=18) cycles. For comparison between the groups, the first COS cycle results of each patient were analyzed.

### Embryo Scoring

The mean embryo score (MES) and the number of good embryos were calculated as parameters for additional analysis in cases of embryo cryopreservation. The scoring methods were adapted from a previous paper (28). Briefly, the score for cleavage-stage embryos was based on the Steer method (29). The total score was calculated by multiplying the cell number by each grading score (A=4, B=, C=2, D=1). The MES was obtained as the total embryo score divided by the total number of embryos produced. Compaction was scored as 40 points and morula was scored as 72 points.

The score for blastocysts was calculated as the development score × the inner cell mass score × the trophectoderm score, based on Gardners’ method (30). The development score was assigned as follows: early blastocyst (ErB) =1; middle expanding blastocyst (MEB) =2; expanded or fully expanded blastocyst=3.5; expanded blastocyst with partial hatching=5; and fully hatched blastocyst=6. The scores for both the inner cell mass and the trophectoderm were assigned separately based on their grade, which were as follows: A=3, B=2, and C=1. The overall scoring method followed that of the previous paper (28).

Regarding the definition of good embryos, for cleavage embryos higher than 6B were assigned, and for blastocysts higher than MEB-BB were assigned. There was no severe oligoasthenoteratozoospermia when IVF or ICSI was performed because of a male factor among infertile patients.

### Outcome Measure

The primary outcomes of the study were the total number of retrieved oocytes, the number of mature oocytes, and the number of cryopreserved oocytes. The MES and the number of good embryos were calculated for secondary analysis in embryo cryopreservation. These indicators were compared within patients with endometrioma according to laterality and between patients with endometrioma and infertile patients or patients with benign cysts. For women who underwent repeated COS, the number of oocytes and mature oocytes collected each time and the cumulative number were scored.

### Statistical Analysis

All statistical analyses were performed using R version 4.0.1 (R Core Team, 2020). Data are presented as median [interquartile range (IQR)] and categorical variables are presented as “n (%)” in the tables. Mann-Whitney-U test was used for continuous variables, and Fisher’s exact test was used for categorical variables. For comparison of infertile patients with patients with endometrioma, propensity score matching by age and BMI was used. With propensity score matching, linear regression analysis was further performed between the patients with a benign ovarian cyst and those with endometrioma due to the small number of patients. For the analysis of the repeated cycle, a generalized estimating equation was used, and the graph was generated using GraphPad Prism version 5.0 (Graph-Pad, San Diego, CA, USA). P <0.05 was considered statistically significant.

## Results

A total of 62 women with endometrioma underwent FP treatment. Of these patients, 50 underwent oocyte cryopreservation and 12 chose embryo cryopreservation. Ninety-five COS cycles were performed in patients with endometrioma. The basal characteristics of the patients who underwent FP (either oocyte or embryo cryopreservation) are presented in **Table 1** according to endometrioma laterality. There was no difference in basal characteristics with respect to endometrioma laterality. The outcomes of the 95 FP COS cycles are described in **Table 2**. The maximum number of cycles performed in a single patient was 4, and 34.7% of cases were treated with COS more than once. The median number of retrieved oocytes was 5.0, the median number of retrieved mature oocytes was 3.0, and the maturation rate was 58.3%. These values did not differ according to laterality. The histologic report confirmed endometriotic cyst.

**Table 1:** Basal characteristics of the patients with endometrioma according to laterality.

Variables	Total	Unilateral	Bilateral	*p* value
	N = 62	N = 34	N = 28	
Age (yr.)^+^	32.5 [27.3, 37.8]	33.0 [27.5, 37.8]	31.0 [27.8, 37.3]	0.843
BMI (kg/m^2^)^+^	21.3 [19.8, 22.7]	21.5 [19.6, 22.8]	21.3 [20.2, 22.6]	0.901
Basal FSH (mIU/mL)^+^	6.7 [5.0, 8.1]	6.8 [6.1, 8.9]	5.8 [4.6, 7.5]	0.222
AMH (ng/mL)^+^	1.2 [0.8, 2.2]	1.2 [0.7, 2.2]	1.4 [1.0, 2.2]	0.144
Previous ovarian surgery before COS^#^				0.999
No	36 (58.1%)	20 (58.8%)	16 (57.1%)	
Yes	26 (41.9%)	14 (41.2%)	12 (42.9%)	
Diameter of largest cyst at diagnosis (cm)^+^	5.2 [3.3, 6.6]	4.3 [3.0, 6.2]	5.6 [4.4, 7.4]	0.091

^+^Mann-Whitney-U test.

^#^Fisher’s exact test.

Data are presented as number (%), or Median [IQR].

BMI, body mass index; COS, controlled ovarian stimulation.

**Table 2:** Controlled ovarian stimulation outcomes of the patients with endometrioma according to endometrioma laterality.

Variables	Total	Unilateral	Bilateral	*p* value
	N = 95	N = 53	N = 42	
FP cycles^#^				0.471
First	62 (65.3%)	34 (64.2%)	28 (66.7%)	
Second	22 (23.2%)	12 (22.6%)	10 (23.8%)	
Third	8 (8.4%)	4 (7.5%)	4 (9.5%)	
Fourth	3 (3.1%)	3 (5.7%)	0 (0%)	
Total dose of gonadotropins (IU)^+^	2400.0 [2100.0, 2700.0]	2400.0 [2400.0, 2700.0]	2400.0 [2100.0, 2700.0]	0.115
Duration of stimulation (day)^+^	8.0 [8.0, 9.0]	8.0 [8.0. 9.0]	8.0 [7.0. 9.0]	0.470
Peak serum E2 levels (pg/mL)^+^	827.0 [521.0, 1859.0]	903.0 [614.0, 2270.0]	750.5 [376.0, 1624.25]	0.073
Number of oocytes retrieved (n)^+^	5.0 [3.0, 8.0]	5.0 [3.0, 8.0]	4.5 [3.0, 7.0]	0.684
Number of mature oocytes retrieved (n)^+^	3.0 [1.5, 5.0]	3.0 [2.0, 5.0]	3.0 [1.0, 4.8]	0.425
Percentage of mature oocytes (%)^+^	58.3 [50.0, 85.7]	60.0 [50.0, 88.9]	57.7 [50.0, 75.0]	0.817
**Variables**	**Total** **N = 18**	**Unilateral** **N = 15**	**Bilateral** **N = 3**	***p* value**
Mean embryo score (MES)*	23.7 [19.3, 31.5]	27.4 [22.2, 32.3]	15.7 [14.8, 17.2]	0.035
Number of good embryo (n)*	2.0 [1.0, 3.0]	2.0 [1.5, 3.0]	1.0 [1.0, 1.5]	0.145
Percentage of good embryos (%)*	100.0 [68.8, 100.0]	100.0 [92.9, 100.0]	33.3 [27.8, 33.3]	0.004

^+^Mann-Whitney-U test.

^#^Fisher’s exact test.

Data are presented as number (%), or Median [IQR].

E2, estradiol.

*These data were analyzed only for patients with embryo cryopreservation.

In case analysis only for embryo cryopreservation (18 cycles), the unilateral group showed significantly better MES than the bilateral group (27.4 [22.2, 32.3] vs. 15.7 [14.8, 17.2], p=0.035), and the rate of good embryo generation was also higher in the unilateral group (100.0 [92.9, 100.0] vs. 33.3 [27.8, 33.3], p=0.004). However, the number of good embryos in each group did not differ significantly (**Table 2**).

**Table 3** shows a comparison of clinical characteristics and cycle outcomes of the first COS cycle in the patients with endometrioma and infertile patients by propensity score matching. Considering that the same age was matched, basal AMH levels were significantly lower in patients with endometrioma than in infertile patients without endometrioma, and the same results were also found in the total number of acquired oocytes (1.2 [0.8, 2.2] vs. 3.3 [2.2, 3.9], p<0.001; 4.0 [2.0, 8.0] vs. 7.0 [5.0, 12.0], P=0.009, respectively). Contrarily, the amount of gonadotropin used was significantly higher in endometrioma patients compared to the amount used in infertile patients (2400.0 [2100.0, 2400.0] vs. 1950.0 [1725.0, 2400.0], p<0.001). Regarding the number of retrieved mature oocytes, infertile patients showed higher median values than patients with endometrioma, but no statistical difference was noted (4.0 [2.0, 7.0] vs. 3.0 [1.0, 5.0], p=0.085). In addition, the stimulation duration and the peak E2 level did not differ between the two groups.

**Table 3:** Comparison of clinical characteristics and first controlled ovarian stimulation cycle outcomes of patients with endometrioma and infertile patients without endometrioma.

Variables	Total	Patients with endometrioma	Infertile patients	*p* value
	N = 86	N = 43	N = 43	
Age (year)	34.0 [31.0, 37.0]	34.0 [30.0, 38.0]	34.0 [32.0, 36.5]	0.581
BMI (kg/m^2^)	21.6 [20.0, 23.1]	21.5 [20.1, 22.8]	22.2 [20.1, 23.8]	0.213
Basal FSH (mIU/mL)	6.1 [4.8, 7.7]	6.3 [4.8, 7.6]	5.8 [4.9, 7.1]	0.359
AMH (ng/mL)	2.2 [1.2, 3.4]	1.2 [0.8, 2.2]	3.3 [2.2, 3.9]	<0.001
Total dose of gonadotropins (IU)	2100.0 [1800.0, 2400.0]	2400.0 [2100.0, 2400.0]	1950.0 [1725.0, 2400.0]	<0.001
Duration of stimulation (day)	8.0 [7.0, 8.0]	8.0 [7.0, 8.0]	8.0 [7.0, 9.0]	0.841
Peak serum E2 levels (pg/mL)	1483.5 [704.0, 2241.8]	907.0 [511.5, 1829.0]	1567.0 [773.0, 2408.0]	0.346
Number of oocytes retrieved (n)	6.0 [3.0, 11.0]	4.0 [2.0, 8.0]	7.0 [5.0, 12.0]	0.009
Number of mature oocytes retrieved (n)	3.0 [2.0, 6.0]	3.0 [1.0, 5.0]	4.0 [2.0, 7.0]	0.085
Percentage of mature oocytes (%)	60.0 [41.4, 80.0]	50.0 [42.7, 85.7]	60.0 [43.2, 73.2]	0.706

All data were analyzed with Mann-Whitney U test. Data are presented as Median [IQR].

BMI, body mass index.

E2, estradiol.

**Table 4** shows the clinical characteristics and outcomes of the first COS cycle in patients with endometrioma and in patients with a benign cyst. There was no statistically significant difference, but the results of AMH, peak E2 level, total number of acquired oocytes, and acquired mature oocytes were lower in the endometrioma group than those in the benign cyst group (1.2 [0.8, 2.2] vs. 1.9 [1.2, 2.6], p=0.094; 832.0 [498.0, 1829.0] vs. 1506.0 [1302.0, 2248.0], p=0.050; 5.0 [3.0, 8.0] vs. 7.5 [3.8, 10.0], p=0.279; 3.0 [1.3, 4.8] vs. 5.5 [2.8, 8.0], p=0.052, respectively). The histologic report confirmed benign ovarian cysts such as mature cystic teratoma, mucinous cystadenoma or fibroma.

**Table 4:** Comparison of clinical characteristics and first controlled ovarian stimulation cycle outcomes of patients with endometrioma and patients with a benign ovarian cyst.

Variables	Total	Patients with endometrioma	Patients with benign cyst	*p* value
	N = 42	N = 21	N = 21	
Basal FSH (mIU/mL)	6.5 [4.9, 7.8]	6.7 [5.0, 8.1]	6.7 [5.1, 8.6]	0.953
AMH (ng/mL)	1.4 [0.9, 2.2]	1.2 [0.8, 2.2]	1.9 [1.2, 2.6]	0.094
Total dose of gonadotropins (IU)	2400.0 [2100.0, 2400.0]	2400.0 [2100.0, 2700.0]	2400.0 [2081.3, 2475.0]	0.900
Duration of stimulation (day)	8.0 [7.0, 9.0]	8.0 [7.0, 9.0]	8.0 [7.8, 9.0]	0.673
Peak serum E2 levels (pg/mL)	1452.0 [720.3, 2187.5]	832.0 [498.0, 1829.0]	1506.0 [1302.0, 2248.0]	0.050
Number of oocytes retrieved (n)	6.0 [3.0, 9.0]	5.0 [3.0, 8.0]	7.5 [3.8, 10.0]	0.279
Number of mature oocytes retrieved (n)	4.0 [2.0, 6.0]	3.0 [1.3, 4.8]	5.5 [2.8, 8.0]	0.052
Percentage of mature oocytes (%)	69.1 [52.8, 100.0]	59.2 [50.0, 85.7]	73.9 [65.9, 88.1]	0.129

All data were analyzed with Mann-Whitney U test.

Data are presented as Median [IQR].

BMI, body mass index.

E2, estradiol.

**Figure 1:**
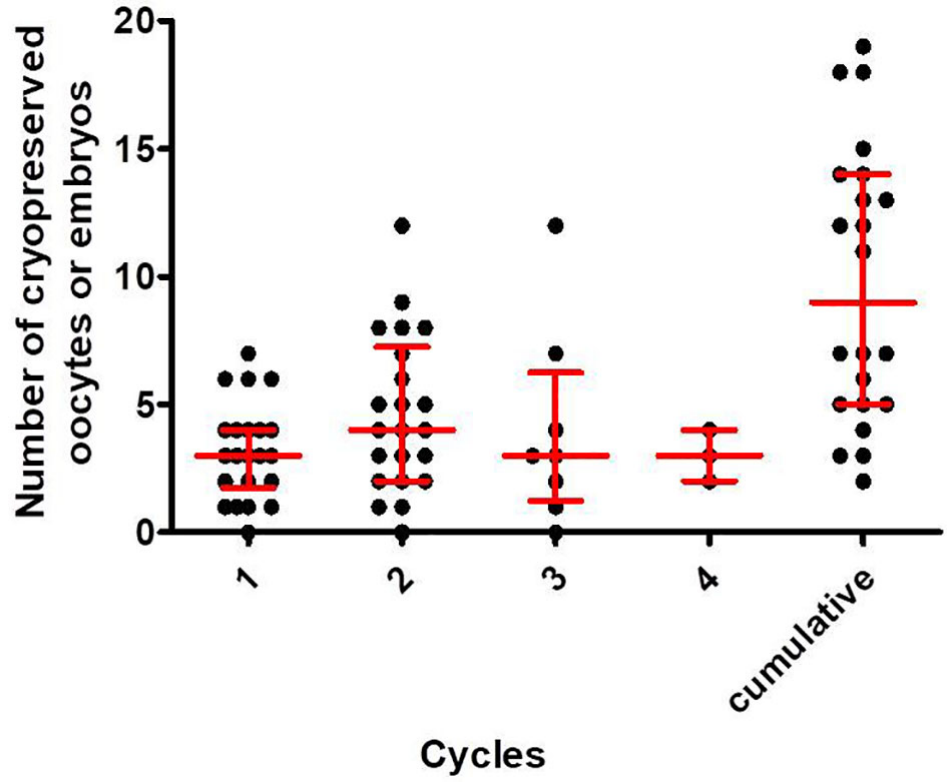
The number of cryopreserved oocytes or embryos in the first, second, third and fourth cycles and the total cumulative numbers in each patient who underwent two or more COS cycles. The horizontal line in each column represents the median value, and the upper and lower bounds represent interquartile ranges.

The repeated COS cycle outcomes are shown in **Table 5**. Even in repeated trials, the number of retrieved oocytes or mature oocytes for each cycle is not decreased, and the median value of the cumulative number of cryopreserved oocytes or embryos was 3 up to the first cycle, 8 up to the second cycle, 12 up to the third cycle, and 14.5 up to the fourth cycle. For patients with two or more repeated COS cycles (n=22, 35.5%), a progressive increase in the number of accumulated cryopreserved oocytes or embryos was noted as the cycles are repeated (**Figure 1**). In this case, the number of cryopreserved oocytes or embryos were not decreased in each cycle. The median [interquartile range] number of cryopreserved oocytes or embryos at final cumulative cycles was 9.0 [5.0, 14.0].

**Table 5:** Cumulative effect of cryopreservation.

	1^st^ cycle	Up to 2^nd^ cycles	Up to 3^rd^ cycles	Up to 4^th^ cycles
	N=62	N=22	N=8	N=3
Number of retrieved oocytes per cycle*	5.0 [3.0, 8.0]	6.0 [3.3, 7.8]	3.5 [3.0, 7.0]	5.0 [4.5, 5.5]
Number of mature oocytes per cycle*	3.0 [1.3, 4.8]	3.0 [1.3. 5.0]	2.5 [1.8, 3.8]	3.0 [2.5, 3.5]
Number of cryopreserved oocytes or embryos per cycle*	3.0 [2.0, 6.0]	4.5 [2.8, 7.3]	3.0 [2.5, 5.5]	3.0 [2.5, 3.5]
Number of cumulative oocytes	5.0 [3.0, 8.0]	10.0 [6.0, 14.5]	16.0 [8.0, 19.8]	21.0 [16.5, 21.0]
Number of cumulative mature oocytes	3.0 [1.3, 4.8]	6.5 [3.5, 9.8]	9.0 [6.8, 11.5]	13.0 [10.0, 13.0]
Number of cumulative cryopreserved oocytes or embryos	3.0 [2.0, 6.0]	8.0 [5.0, 12.0]	12.0 [9.0, 16.0]	14.5 [14.3, 14.8]

Data are presented as Median [IQR].

*The median number of oocytes or embryos in each cycle (1st, 2nd, 3rd, and 4th) is not statistically different (generalized estimating equation).

## Discussion

The present study demonstrated that AMH level was significantly lower in patients with endometrioma than in infertile patients of the same age, and fewer oocytes were obtained. Although there was no significant difference, the same trend was confirmed when comparisons were made with the benign ovarian cyst group. Patients with bilateral endometrioma showed significantly lower MES and percentage of good embryos than patients with unilateral endometrioma. Preoperative oocyte or embryo cryopreservation can successfully preserve fertility, and through repeated COS, a larger number of cumulative oocytes or embryos can be cryopreserved for future fertility without a decrease in the number of collected oocytes.

In this study, we did not observe any significant difference in COS outcomes according to endometrioma laterality. In a previous study, it was reported that the decrease in ovarian reserve was significantly more in bilateral ovarian cystectomy (BOC) than that in unilateral ovarian cystectomy (UOC) (8). Therefore, it can be said that the need for FP is greater in BOC. Considering that there was no significant difference in the preoperative COS outcomes between BOC and UOC groups, FP treatment before ovarian cystectomy, especially in women with bilateral endometrioma is much more important.

Regarding the results of embryo cryopreservation, the quality of the embryos in the bilateral endometrioma group was significantly lower than that in the unilateral group. It can be considered that the probability of generating oocytes of normal quality was lowered, possibly resulting in lower quality embryos being generated. It was reported that the quality of the ovarian follicle adjacent to the endometrioma was damaged, atretic, and had a low mitochondrial content (3, 31). Mechanical stretching, decrease in granulosa cell function, increase in reactive oxygen species and gonadotoxic free iron (31), inflammatory cytokines in follicular fluid (32), formation of abnormal meiotic spindle and chromosomal misalignment (33), and polar body abnormality (34) were suggested as possible mechanisms of deterioration in oocyte quality.

Compared to the benign cyst group, patients with endometrioma showed lower peak E2 level with marginal significance (p=0.05). Other COS outcomes such as retrieved total and mature oocyte number and oocyte maturation rate showed lower results although the difference did not reach statistical significance. Endometrioma could affect the adjacent ovarian follicle and stroma and cause detrimental outcomes (31). The worse COS outcomes could be expected in women with endometrioma and less oocytes could be obtained compared to those with benign cyst. The necessity of repeated COS could be explained to the patients.

In comparison with the infertile patient group of the same age, the endometrioma group showed significantly lower AMH and oocyte yield. Patients with endometrioma showed a faster decrease in preoperative AMH level compared to age-matched healthy patients (35). This was especially notable in bilateral cases (36). This finding is consistent with the results of a previous study that showed a greater decline in AMH levels in the endometrioma group, and this trend was maintained after surgery (37). Therefore, an active medical or surgical measure to prevent disease progression is essential in patients with endometriosis. If surgical treatment is required, deterioration of the ovarian reserve will follow after surgery due to the accompanying removal of the normal ovarian tissue or injury to the ovarian vasculature (3, 38). In the case of bilateral (39) or recurrent surgery, the reduction is more prominent than in the initial surgery (40); thus, the procedure for FP before surgery must be performed.

In women with endometrioma, lower AMH and poor COS outcomes were observed compared to those in the infertile control group, resulting in a small number of oocytes retrieved per cycle. However, multiple COS cycles are feasible in patients with endometrioma with patient agreement, because there is no urgent treatment such as chemotherapy in cancer patients. In the analysis of our study a median of up to 14.5 oocytes or embryos with 4 COS trials could be cryopreserved. Therefore, it is necessary to counsel patients that even if a small number of oocytes was obtained in the first cycle, sufficient oocytes or embryos for future pregnancy attempts could be achieved with multiple repeats of the procedure. Our center recommends that at least 10–15 oocytes should be cryopreserved in consideration of the future pregnancy if the patient is willing to undergo FP. Considering the probability of live birth, Struli et al. suggested cryopreserving 15–20 oocytes in women under 38 years and 25–30 oocytes in women under 38–40 years (41). Based on these findings, the number of oocytes that we recommend seems to be small. However, ovarian reserve test will be followed up regularly after surgery and further FP will be performed if necessary. Concerns about increasing size of endometrioma could be raised for the administration of exogenous hormones during COS. However, studies that performed COS for infertility treatment have shown that there was no further increase in the recurrence of endometriosis (42, 43).

There is still no consent recommendation for FP in patients with endometrioma (5, 41). Limitedly, Natalia C et al. recommended that FP may be offered on an individual basis to young women at high risk of endometriosis recurrence, or those at risk of damage to the bilateral ovaries (1). A working group of the European Society of Human Reproduction and Embryology (ESHRE), and the World Endometriosis Society (WES) recommend awareness of the risk of damage to ovarian reserve in endometrioma surgery, and FP should be considered when the reserve is already compromised (44). However, at present, information on clear indications and standard tests are not distinct, and ESHRE guidelines (45) limits the value of AMH for FP in patients with endometriosis. Therefore, establishment of indications for patient groups that benefit from FP, and additional validation work are needed in the future. Personalized counseling that considers age, involvement of endometriosis, and current ovarian reserve should be provided. In addition, in cases where oocyte or embryo cryopreservation is not affordable, ovarian tissue cryopreservation could be discussed and considered as a FP option (3).

The strength of the present study is that we analyzed and verified a previous pilot study conducted by us, we enrolled more patients, and compared other patient groups, such as patients with infertility or those with cysts other than endometrioma. However, the retrospective design of the study and the unavailability of pregnancy results are two major limitations of this study. In summary, elective FP before ovarian surgery can preserve fertility for women with endometrioma with good feasibility, and increasing the number of accumulated cryopreserved oocytes or embryos with repeated cycles can provide higher chance of future fertility.

## Data Availability Statement

The raw data supporting the conclusions of this article will be available on reasonable request to the corresponding author.

## Ethics Statement

The studies involving human participants were reviewed and approved by the Institutional Review Board of the Seoul National University Bundang Hospital (No. B-2006/616-108). Written informed consent for participation was not required for this study in accordance with the national legislation and the institutional requirements.

## Author Contributions

YH and JL: design of the study and the manuscript writing. YH, HL, SK, JL, and CS: gathering and analyzing the data. All authors: discussion of the results and revision of the manuscript. All authors contributed to the article and approved the submitted version.

## Funding

This research was supported by a grant of the Korea Health Technology R&D Project through the Korea Health Industry Development Institute (KHIDI), funded by the Ministry of Health & Welfare, Republic of Korea (grant number. HI21C1353).

## Conflict of Interest

The authors declare that the research was conducted in the absence of any commercial or financial relationships that could be construed as a potential conflict of interest.

## Publisher’s Note

All claims expressed in this article are solely those of the authors and do not necessarily represent those of their affiliated organizations, or those of the publisher, the editors and the reviewers. Any product that may be evaluated in this article, or claim that may be made by its manufacturer, is not guaranteed or endorsed by the publisher.

## References

[1] Llarena NC Falcone T Flyckt RL. Fertility Preservation in Women With Endometriosis. J Clin Med Insights: Reprod Health (2019) 13:1179558119873386. doi: 10.1177/1179558119873386 PMC6724494 31516316

[2] Practice Committee of the American Society for Reproductive Medicine, Endometriosis and Infertility: A Committee Opinion. Fertil Steril (2012) 98(3):591–8. doi: 10.1016/j.fertnstert.2012.05.031 22704630

[3] Llarena NC Flyckt RL. Strategies to Preserve and Optimize Fertility for Patients With Endometriosis. J Endometriosis Pelvic Pain Disord (2017) 9(2):98–104. doi: 10.5301/jeppd.5000278

[4] Kitajima M Dolmans M-M Donnez O Masuzaki H Soares M Donnez JJF. Enhanced Follicular Recruitment and Atresia in Cortex Derived From Ovaries With Endometriomas. Fertil Steril (2014) 101(4):1031–7. doi: 10.1016/j.fertnstert.2013.12.049 24502890

[5] Somigliana E Viganò P Filippi F Papaleo E Benaglia L Candiani M. Fertility Preservation in Women With Endometriosis: For All, for Some, for None? Hum Reprod (2015) 30(6):1280–6. doi: 10.1093/humrep/dev078 25883035

[6] Celik HG Dogan E Okyay E Ulukus C Saatli B Uysal S. Effect of Laparoscopic Excision of Endometriomas on Ovarian Reserve: Serial Changes in the Serum Antimüllerian Hormone Levels. Fertil Steril (2012) 97(6):1472–8. doi: 10.1016/j.fertnstert.2012.03.027 22521696

[7] Hirokawa W Iwase A Goto M Takikawa S Nagatomo Y Nakahara T. The Post-Operative Decline in Serum Anti-Müllerian Hormone Correlates With the Bilaterality and Severity of Endometriosis. Hum Reprod (2011) 26(4):904–10. doi: 10.1093/humrep/der006 21292639

[8] Chang HJ Han SH Lee JR Jee BC Lee BI Suh CS. Impact of Laparoscopic Cystectomy on Ovarian Reserve: Serial Changes of Serum Anti-Müllerian Hormone Levels. Fertil Steril (2010) 94(1):343–9. doi: 10.1016/j.fertnstert.2009.02.022 19345350

[9] Biacchiardi CP Delle Piane L Camanni M Deltetto F Delpiano EM Marchino GL. Laparoscopic Stripping of Endometriomas Negatively Affects Ovarian Follicular Reserve Even If Performed by Experienced Surgeons. Reprod BioMed Online (2011) 23(6):740–6. doi: 10.1016/j.rbmo.2011.07.014 22019621

[10] Almog B Shehata F Sheizaf B Tulandi T. Effect of Different Types of Ovarian Cyst on Antral Follicle Count. Fertil Steril (2010) 94(6):2338–9. doi: 10.1016/j.fertnstert.2010.01.074 20226445

[11] Younis JS Shapso N Fleming R Ben-Shlomo I Izhaki I. Impact of Unilateral Versus Bilateral Ovarian Endometriotic Cystectomy on Ovarian Reserve: A Systematic Review and Meta-Analysis. Hum Reprod Update (2019) 25(3):375–91. doi: 10.1093/humupd/dmy049 30715359

[12] De Vos M Smitz J Woodruff TK. Fertility Preservation in Women With Cancer. Lancet (2014) 384(9950):1302–10. doi: 10.1016/S0140-6736(14)60834-5 PMC4270060 25283571

[13] Elizur SE Chian R-C Holzer HE Gidoni Y Tulandi T Tan SL. Cryopreservation of Oocytes in a Young Woman With Severe and Symptomatic Endometriosis: A New Indication for Fertility Preservation. Fertil Steril (2009) 91(1):e1–.e3:293. doi: 10.1016/j.fertnstert.2007.06.040 17920595

[14] Lantsberg D Fernando S Cohen Y Rombauts L. The Role of Fertility Preservation in Women With Endometriosis: A Systematic Review. J Minim Invasive Gynecol (2020) 27(2):362–72. doi: 10.1016/j.jmig.2019.09.780 31546067

[15] Donnez J Squifflet J Dolmans M-M Martinez-Madrid B Jadoul P Van Langendonckt A. Orthotopic Transplantation of Fresh Ovarian Cortex: A Report of Two Cases. Fertil Steril (2005) 84(4):1018.e1–3. doi: 10.1016/j.fertnstert.2005.06.011 16213862

[16] Schubert B Canis M Darcha C Artonne C Pouly J-L Déchelotte P. Human Ovarian Tissue From Cortex Surrounding Benign Cysts: A Model to Study Ovarian Tissue Cryopreservation. Hum Reprod (2005) 20(7):1786–92. doi: 10.1093/humrep/dei002 15802317

[17] Raad J Sonigo C Tran C Sifer C Durnerin IC Grynberg M. Oocyte Vitrification for Preserving Fertility in Patients With Endometriosis: First Observational Cohort Study … and Many Unresolved Questions. Letter to the Editor. Eur J Obstet Gynecol Reprod Biol (2018) 220:140–1. doi: 10.1016/j.ejogrb.2017.12.001 29221758

[18] Kuroda K Ikemoto Y Ochiai A Ozaki R Matsumura Y Nojiri S. Combination Treatment of Preoperative Embryo Cryopreservation and Endoscopic Surgery (Surgery-ART Hybrid Therapy) in Infertile Women With Diminished Ovarian Reserve and Uterine Myomas or Ovarian Endometriomas. J Minim Invasive Gynecol (2019) 26(7):1369–75. doi: 10.1016/j.jmig.2019.02.008 30794888

[19] Garavaglia E Sala C Taccagni G Traglia M Barbieri C Ferrari S. Fertility Preservation in Endometriosis Patients: Anti-Müllerian Hormone Is a Reliable Marker of the Ovarian Follicle Density. Front Surg (2017) 4:40. doi: 10.3389/fsurg.2017.00040 28791295 PMC5524724

[20] Garcia-Velasco JA Domingo J Cobo A Martínez M Carmona L Pellicer A. Five Years’ Experience Using Oocyte Vitrification to Preserve Fertility for Medical and Nonmedical Indications. Fertil Steril (2013) 99(7):1994–9. doi: 10.1016/j.fertnstert.2013.02.004 23465707

[21] Kim SJ Kim SK Lee JR Suh CS Kim SH. Oocyte Cryopreservation for Fertility Preservation in Women With Ovarian Endometriosis. Reprod BioMed Online (2020) 40(6):827–34. doi: 10.1016/j.rbmo.2020.01.028 32295746

[22] Cobo A Giles J Paolelli S Pellicer A Remohí J García-Velasco JA. Oocyte Vitrification for Fertility Preservation in Women With Endometriosis: An Observational Study. Fertil Steril (2020) 113(4):836–44. doi: 10.1016/j.fertnstert.2019.11.017 32145929

[23] d’Argent EM Ferrier C Zacharopoulou C Ahdad-Yata N Boudy A-S Cantalloube A. Outcomes of Fertility Preservation in Women With Endometriosis: Comparison of Progestin-Primed Ovarian Stimulation Versus Antagonist Protocols. J Ovarian Res (2020) 13(1):1–7. doi: 10.1186/s13048-020-00620-z PMC7020543 32054493

[24] Exacoustos C Manganaro L Zupi E. Imaging for the Evaluation of Endometriosis and Adenomyosis. Best Pract Res Clin Obstet Gynaecol (2014) 28(5):655–81. doi: 10.1016/j.bpobgyn.2014.04.010 24861247

[25] Foti PV Farina R Palmucci S Vizzini IAA Libertini N Coronella M. Endometriosis: Clinical Features, MR Imaging Findings and Pathologic Correlation. Insights Into Imaging (2018) 9(2):149–72. doi: 10.1007/s13244-017-0591-0 PMC5893487 29450853

[26] Choi N Kim CK Park BK. US Diagnosis of Mature Cystic Teratomas of the Ovary: Morphologic Analysis of 112 Pathologically Proven Cases. Ultrasonography (2006) 25(4):179–84.

[27] Andrade Neto F Palma-Dias R. Costa FdS. Ultrasonography of Adnexal Masses: Imaging Findings. Radiol Bras (2011) 44:59–67. doi: 10.1590/S0100-39842011000100014

[28] Hong YH Lee JM Kim SK Youm HW Jee BC. Associations of Post-Warming Embryo or Blastocyst Development With Clinical Pregnancy in Vitrified Embryo or Blastocyst Transfer Cycles. Clin Exp Reprod Med (2020) 47(2):140. doi: 10.5653/cerm.2019.03321 32456411 PMC7315863

[29] Steer C Mills C Tan S Campbell S Edwards R. The Cumulative Embryo Score: A Predictive Embryo Scoring Technique to Select the Optimal Number of Embryos to Transfer in an *In-Vitro* Fertilization and Embryo Transfer Programme. Hum Reprod (1992) 7(1):117–9. doi: 10.1093/oxfordjournals.humrep.a137542 1551945

[30] Schoolcraft WB Gardner DK Lane M Schlenker T Hamilton F Meldrum DR. Blastocyst Culture and Transfer: Analysis of Results and Parameters Affecting Outcome in Two *In Vitro* Fertilization Programs. Fertil Steril (1999) 72(4):604–9. doi: 10.1016/S0015-0282(99)00311-8 10521095

[31] Sanchez AM Vanni VS Bartiromo L Papaleo E Zilberberg E Candiani M. Is the Oocyte Quality Affected by Endometriosis? A Review of the Literature. J Ovarian Res (2017) 10(1):1–11. doi: 10.1186/s13048-017-0341-4 28701212 PMC5508680

[32] Singh AK Dutta M Chattopadhyay R Chakravarty B Chaudhury K. Intrafollicular Interleukin-8, Interleukin-12, and Adrenomedullin Are the Promising Prognostic Markers of Oocyte and Embryo Quality in Women With Endometriosis. J Assist Reprod Genet (2016) 33(10):1363–72. doi: 10.1007/s10815-016-0782-5 PMC5065558 27491770

[33] Mansour G Sharma RK Agarwal A Falcone T. Endometriosis-Induced Alterations in Mouse Metaphase II Oocyte Microtubules and Chromosomal Alignment: A Possible Cause of Infertility. Fertil Steril (2010) 94(5):1894–9. doi: 10.1016/j.fertnstert.2009.09.043 19896655

[34] Cohen J Ziyyat A Naoura I Chabbert-Buffet N Aractingi S Darai E. Effect of Induced Peritoneal Endometriosis on Oocyte and Embryo Quality in a Mouse Model. J Assist Reprod Genet (2015) 32(2):263–70. doi: 10.1007/s10815-014-0390-1 PMC4354196 25399065

[35] Kasapoglu I Ata B Uyaniklar O Seyhan A Orhan A Oguz SY. Endometrioma-Related Reduction in Ovarian Reserve (ERROR): A Prospective Longitudinal Study. Fertil Steril (2018) 110(1):122–7. doi: 10.1016/j.fertnstert.2018.03.015 29935810

[36] Nieweglowska D Hajdyla-Banas I Pitynski K Banas T Grabowska O Juszczyk G. Age-Related Trends in Anti-Mullerian Hormone Serum Level in Women With Unilateral and Bilateral Ovarian Endometriomas Prior to Surgery. Reprod Biol Endocrinol (2015) 13(1):1–9. doi: 10.1186/s12958-015-0125-x 26596960 PMC4657379

[37] Chen Y Pei H Chang Y Chen M Wang H Xie H. The Impact of Endometrioma and Laparoscopic Cystectomy on Ovarian Reserve and the Exploration of Related Factors Assessed by Serum Anti-Mullerian Hormone: A Prospective Cohort Study. J Ovarian Res (2014) 7(1):1–8. doi: 10.1186/s13048-014-0108-0 25424986 PMC4255637

[38] Somigliana E Berlanda N Benaglia L Viganò P Vercellini P Fedele L. Surgical Excision of Endometriomas and Ovarian Reserve: A Systematic Review on Serum Antimüllerian Hormone Level Modifications. Fertil Steril (2012) 98(6):1531–8. doi: 10.1016/j.fertnstert.2012.08.009 22975114

[39] Goodman LR Goldberg JM Flyckt RL Gupta M Harwalker J Falcone T. Effect of Surgery on Ovarian Reserve in Women With Endometriomas, Endometriosis and Controls. Am J Obstet Gynecol (2016) 215(5):589. doi: 10.1016/j.ajog.2016.05.029 27242204

[40] Muzii L Achilli C Lecce F Bianchi A Franceschetti S Marchetti C. Second Surgery for Recurrent Endometriomas Is More Harmful to Healthy Ovarian Tissue and Ovarian Reserve Than First Surgery. Fertil Steril (2015) 103(3):738–43. doi: 10.1016/j.fertnstert.2014.12.101 25577464

[41] Streuli I Benard J Hugon-Rodin J Chapron C Santulli P Pluchino N. Shedding Light on the Fertility Preservation Debate in Women With Endometriosis: A Swot Analysis. Eur J Obstet Gynecol Reprod Biol (2018) 229:172–8. doi: 10.1016/j.ejogrb.2018.08.577 30199816

[42] D’Hooghe TM Denys B Spiessens C Meuleman C Debrock S. Is the Endometriosis Recurrence Rate Increased After Ovarian Hyperstimulation? Fertil Steril (2006) 86(2):283–90. doi: 10.1016/j.fertnstert.2006.01.016 16753162

[43] Wu CQ Albert A Alfaraj S Taskin O Alkusayer GM Havelock J. Live Birth Rate After Surgical and Expectant Management of Endometriomas After *In Vitro* Fertilization: A Systematic Review, Meta-Analysis, and Critical Appraisal of Current Guidelines and Previous Meta-Analyses. J Minim Invasive Gynecol (2019) 26(2):299–311.e3. doi: 10.1016/j.jmig.2018.08.029 30717864

[44] Working group of ESGE, ESHRE and WES Saridogan E Becker CM Feki A Grimbizis GF. Recommendations for the Surgical Treatment of Endometriosis. Part 1: Ovarian Endometrioma. Hum Reprod Open (2017) 2017(4):hox016. doi: 10.1093/hropen/hox016 31486802 PMC6277006

[45] Preservation EGGoFF Anderson RA Amant F Braat D D’Angelo A Chuva de Sousa Lopes SM. ESHRE Guideline: Female Fertility Preservation. Hum Reprod Open (2020) 2020(4):hoaa052. doi: 10.1093/hropen/hoaa052 33225079 PMC7666361

